# Moderate aerobic training enhances the effectiveness of insulin therapy through hypothalamic IGF1 signaling in rat model of Alzheimer's disease

**DOI:** 10.1038/s41598-024-66637-2

**Published:** 2024-07-10

**Authors:** Forough Radfar, Mehdi Shahbazi, Shahzad Tahmasebi Boroujeni, Elahe Arab Ameri, Maryam Farahmandfar

**Affiliations:** 1 Department of Behavioral and Cognitive Sciences in Sports, Sports and Health Sciences Faculty, University of Tehran, Tehran, 1417935837 Iran; 2 Electrophysiology Research Center, Neuroscience Institute, Tehran University of Medical Sciences, Tehran, 14177-55469 Iran

**Keywords:** Alzheimer’s disease, Insulin, Metabolism, Spatial learning and memory, Treadmill exercise, Neuroscience, Diseases, Health care

## Abstract

Alzheimer's disease (AD) is a neurological condition that is connected with a decline in a person's memory as well as their cognitive ability. One of the key topics of AD research has been the exploration of metabolic causes. We investigated the effects of treadmill exercise and intranasal insulin on learning and memory impairment and the expression of IGF1, BDNF, and GLUT4 in hypothalamus. The animals were put into 9 groups at random. In this study, we examined the impact of insulin on spatial memory in male Wistar rats and analyzed the effects of a 4-week pretreatment of moderate treadmill exercise and insulin on the mechanisms of improved hypothalamic glucose metabolism through changes in gene and protein expression of IGF1, BDNF, and GLUT4. We discovered that rat given Aβ_25–35_ had impaired spatial learning and memory, which was accompanied by higher levels of Aβ plaque burden in the hippocampus and lower levels of IGF1, BDNF, and GLUT4 mRNA and protein expression in the hypothalamus. Additionally, the administration of exercise training and intranasal insulin results in the enhancement of spatial learning and memory impairments, the reduction of plaque burden in the hippocampus, and the enhancement of the expression of IGF1, BDNF, and GLUT4 in the hypothalamus of rats that were treated with Aβ_25–35_. Our results show that the improvement of learning and spatial memory due to the improvement of metabolism and upregulation of the IGF1, BDNF, and GLUT4 pathways can be affected by pretreatment exercise and intranasal insulin.

## Introduction

Dementia is the primary cause of disability and dependence among older adults^1^. According to the Alzheimer's Association, the degenerative brain condition known as Alzheimer's disease (AD) is responsible for between 60 and 80% of all instances of dementia. The progression of symptoms that make it difficult to accomplish daily tasks, depending on the stage of the illness, includes apathy, depression, impaired communication, confusion, poor judgment, difficulties swallowing and walking, and behavioral changes^2^. Abnormal changes in brain structures, such as atrophy in the hippocampus and the medial temporal gyrus, usually accompany and potentially cause progressive cognitive decline. Because of the buildup of Aβ plaques and neurofibrillary tangles inside neurons, these atrophies are directly linked to the loss of synapses and neurons. These deposits are thought to cause atrophies and the death of neurons due to excitotoxicity processes (excessive stimulation of neurotransmitter receptors in neural membranes), a breakdown in calcium homeostasis, inflammation, and a depletion of energy and neuronal components.

Although the exact mechanisms by which these changes cause cognitive decline are still under debate, In fact, by causing inflammation and oxidative stress, Aβ plaques and tau tangles can directly impact the structural and functional flexibility of the central nervous system. But current research indicates that energy metabolism, particularly glucose hypometabolism, may contribute to or aggravate AD in addition to Aβ plaque and tau tangles^3^. It is common knowledge that glucose is an indispensable component in the maintenance of a number of brain activities, including the production and recycling of neurotransmitters. The hippocampus, hypothalamus, striatum, and insular cortex all showed signs of impaired glucose metabolism. The insular cortex is the area of the brain that shows the first signs of cognitive impairment in Alzheimer's disease, which suggests that a disruption in glucose metabolism was linked to the development of the disease^4,5^. Neuroinflammation is a proven cause of glucose hypometabolism. Neuroinflammation may influence insulin sensitivity and the enzymatic activity of oxidative phosphorylation, both of which are involved in AD. GLUT-related glucose hypometabolism also affects Aβ levels by raising the activity of secretases, which in turn increases the creation and accumulation of Aβ generation as well as tau hyperphosphorylation^6^. In addition to binding to IGF receptors, insulin-like growth factors (IGF) are proteins with a chemical structure that is almost identical to that of insulin. These growth factors play an essential part in the regulation of metabolism and growth, as well as the maintenance of the proliferation of a significant number of cells located in both the periphery and the center. Insulin and IGF regulate physiological, biochemical, cellular, molecular, and behavioral activities via controlling neurobiological processes such as neuromodulation, neurotrophy, neuronal growth, and neuronal survival^7^. Brain-derived neurotrophic factor (BDNF) serves a variety of purposes in the development and plasticity of the brain^8^. It protects neurons in a wide variety of brain regions against damage and malfunction caused by outside factors^9^. Additionally, BDNF plays a crucial role in the hypothalamus circuit that regulates energy balance^10^. GLUT concentrations decrease in the presence of pro-inflammatory markers and insulin resistance. One of the main characteristics of T2DM and dementia pathogenesis is insulin resistance, which is connected with the downregulation of GLUT gene expression^11^.

Regular exercise is now widely recognized as an effective intervention for slowing the progression of disease. This finding is in line with the theory that having a healthy lifestyle strengthens the brain's resistance to the negative effects of aging and may perhaps postpone the beginning of dementia. Several of the processes underpinning the protective effects of exercise on AD have already been explored in other places^12^. Nevertheless, despite the fact that in vitro and in vivo studies strongly indicate the involvement of Aβ and tau regulation in AD, no treatment management has been proved to be beneficial in human beings. In order to find a solution to this issue, the research presented here offers a fresh point of view. The goal of this paper is to discuss the neuroprotective effects of moderate training exercise and insulin treatment against Alzheimer's disease (AD). These effects include a synergistic improvement in brain glucose metabolism through increasing the level of IGF1, BDNF, and GLUT4 proteins involved in glucose metabolism, as well as an improvement in AD pathophysiology (for example, a reduction in p-tau and Aβ load). Enhancing the expression of IGF1, BDNF, and GLUT4 in the hypothalamus could be a novel strategy for reversing neurodegenerative processes.

## Materials and methods

The current study aims to show that moderate aerobic training enhances the effectiveness of insulin therapy through hypothalamic IGF1 signaling in the rat model of Alzheimer's disease. It has received approval from the ethics committee of the University of Tehran (https://ethics.research.ac.ir/IR.UT.SPORT.REC.1400.004) and adheres to the guidelines and regulations outlined by the scientific reports journal's editorial and publishing policies. We carried out all methods in accordance with the appropriate guidelines and regulations.

### Animals

**Figure 1:**
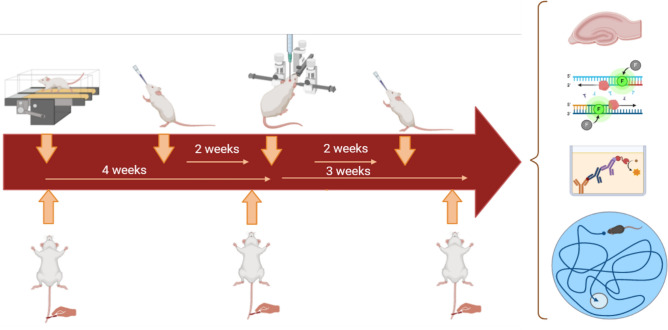
Schematic diagram of the experimental protocol. Aβ (10 µg/rat)/saline were administered bilaterally Intra-cerebroventricular to the animals. Each group received its own training and treatment protocol. And 21 days after the Aβ injection in all groups, learning and spatial memory were evaluated with the MWM test, thioflavin-S, real-time PCR, and ELISA tests.

The procedures were authorized by the Ethics Committee of the University of Tehran in accordance with the ARRIVE guidelines (https://ethics.research.ac.ir/IR.UT.SPORT.REC.1400.004). The University of Tehran's Ethics Committee authorized the procedures, following the ARRIVE guidelines (https://ethics.research.ac.ir/IR.UT.SPORT.REC.1400.004). We obtained male Wistar rats (n = 126; age = 8 weeks) from the Animals Center of the Royan Institute of Iran. We maintained the animals in animal houses with a 12-h light/dark cycle (lights on at 7 a.m. and off at 7 p.m.), providing them with free access to food and water in groups of three per cage. The Wistar rats were randomly put into 9 groups, with 14 rats in each: (1) the healthy control (control); (2) the Sham; (3) the injection beta amyloid (A^2^); (4) the injection beta amyloid + moderate training exercise (A^2^ + EXE); (5) the injection beta amyloid + pretreatment insulin (A^2^ + PIN); (6) the injection beta amyloid + insulin treatment (A^2^ + INT); (7) the injection beta amyloid + moderate training exercise + pretreatment insulin (A^2^ + EXE + PIN); and (8) the injection beta amyloid + moderate training ex Fig. 1 shows a diagrammatic representation of the many processes that were involved in our research. We made every effort to reduce the number of animals utilized and their suffering.

### Aβ (25–35) preparation and intra-cerebroventricular injection

Aβ_25-35_ peptide was administered in order to bring on Alzheimer's disease. To allow peptide fibrillization and aggregation associated with toxicity to occur, an Aβ_25-35_ peptide (Tocris- 131602-53-4, Bristol, UK) was incubated in sterile saline at a concentration of 5 µg/ mL at 37 °C for 72 h. After incubation, the peptide was stored at a temperature of − 20 °C until it was used^13^. Rats were put on a stereotaxic frame after being anesthetized with ketamine and xylazine (70 vs. 10 mg/kg body weight). The intra-cerebroventricular region (ICV: AP: − 0.08, ML: 1.5, DV: 3.5 mm) was implanted bilaterally with a stainless steel guide cannula (23G, 9 mm length)^13^. After a week had passed, the open field task was carried out. Both the sham-operated and the Aβ-treated groups were given bilateral intracerebral injections of either saline or Aβ_25-35_ (10 µg/rat) using an injection needle with a gauge of 27 and a syringe with a volume of 25 ML (Hamilton, Reno, Nevada)^14^.

### Moderate training exercise protocol

Rats were acclimated to their new surroundings and made familiar with the treadmill apparatus by walking at a speed of 5 m per minute for 10 min per day over the course of five days prior to the start of the treadmill training. For four weeks, the exercise routine was carried out five days per week between 9:00 a.m. and 16:00 p.m. on a 0% gradient^15^. The rats ran for 30 min daily (2 × 15-min sessions) at a speed of 10 m/min throughout the first and second weeks. 5 min of inactive rest were allowed in between sessions to prevent muscular fatigue. During the subsequent weeks, the intensity of the exercise as well as the duration of it gradually increased. For example, during the third week, the rats ran for 45 min per day at a speed of 15 m per minute (3 sessions of 15 min each), and during the fourth week, the rats ran for 60 min per day at a speed of 15 m per minute (4 sessions of 15 min each). During the training, the rats were given a moderate shock with an intensity of 0.5 millamperes, which did not cause the animal any undue stress. This was done in order to encourage the rats to run continually^16^.

### Insulin treatment

In a prior investigation, the dose of intranasal insulin that significantly improved memory functions was determined to be 2 IU^17^. The rats' heads were pulled back with a pad while being held by the backs of their necks, and insulin was administered via each nostril using an Eppendorf pipette. Groups 5 and 7 received insulin 14 days before beta injection, groups 6 and 8 received insulin 14 days after beta injection, and group 9 rats received insulin 28 days (14 days before and 14 days after) following a 2 IU beta injection.

### Behavioral assessment

The rats were put through the Morris Water Maze (MWM) test so that researchers could measure their capacity for spatial learning and memory. The pool had a circumference of 150 cm and a height of 90 cm, and it was filled with water at a temperature of 25 °C to a depth of 35 cm. This pool was separated into four equal quadrants, and the center of the south-east quadrant (target quadrant) had a hidden platform constructed of Plexiglas that was 10 cm in diameter and was situated 1 cm below the surface of the water. The test of spatial acquisition was performed over the course of four consecutive days (each animal participated in one block of four trials per day). In each of the trials, a different animal was chosen at random and placed in the wall of the pool at one of the four beginning points of a quadrant. The animal was then given sixty seconds to find the concealed platform. The rats that found the platform within this time frame were permitted to remain on it for a further 20 s, whereas the rats that did not find the platform within the first 60 s were coaxed to it and given permission to relax there for an additional 20 s. After the completion of the phase of acquisition, the probing test was carried out twenty-four hours later. The platform was removed from the water, and after that, each rat was put into the water in the quadrant of the water that was opposite to the objective, and they were given sixty seconds to swim around freely. The level of retention of spatial information was determined by the percentage of time spent in the target quadrant. In order to determine the level of visual-motor coordination possessed by rats, a visible platform task consisting of one block of four separate trials was carried out one hour following the last probe test. The platform was coated with a shiny piece of aluminum foil, raised slightly above the water level (about 1.5 cm), and positioned in the quadrant of the map that was opposite the target (the southwest quadrant). The amount of time that was spent and the distance that was traveled in order to locate the visible platform were both recorded, and the swimming speed of rats during all of the trials was examined. A measurement was taken of how much time the patient spent looking in each of the four quadrants. The Ethovision tracking device was used to assess the escape delay, swimming speed, distance traveled, and amount of time spent within the target zone (during acquisition and the probe test) (version 11.5, Noldus, Netherlands). Calculations and statistical analysis were done with the data that was acquired^18^.

### Preparation of FITC-INS

Fluorescein isothiocyanate-labeled INS (FITC-IN) was created by adding FITC solution in DMSO (5 mg/mL) dropwise while gently stirring IN solution (15 mg/mL) in the dark. The reaction mixture was incubated at room temperature for 30 min, followed by 1 h. Using a PD-10 SephadexTM G25 column, unbound FITC was separated from INS and FITC-IN conjugates, and FITC-INS was lyophilized overnight. The identical procedure outlined above was used to prepare the FITC-IN- loaded TRA(35)^19^.

### Optical imaging study

The use of fluorescence imaging was carried out in order to verify the delivery and distribution of INS in specific regions of the brain and to investigate the course followed by IN as it moved through the body's other organs. In order to investigate how FITC-IN was delivered to the olfactory region of the brain, optical imaging was utilized. One rat was chosen for administration of the FITC-containing systems, and a second rat served as a control to gauge the body's autofluorescence. After thoroughly shaving the rats, 10 mL of FITC-IN was given to them intranasally at the same FITC dose (100 ng FITC/1 g protein). A KODAK imaging system (system FX Pro) was used for in vivo imaging at the Tehran University of Medical Sciences' Preclinical Core Facility (TPCF). With fluorescent mode and a one-minute exposure time, the excitation and emission filters were adjusted to 510 nm and 535 nm, respectively. The KODAK camera system picked up the light the rats were emitting, combined it, digitalized it, and then presented it. Rats underwent optical imaging at 15 min, 1, and 4 h following nasal injection. Live animal autofluorescence is hampered by the short fluorescent wavelength of FITC. This autofluorescence is dramatically decreased in the rats after total shaving^20,21^. In addition to this, imaging of the brain and other key organs was carried out to validate the findings of the live imaging. After four hours, the rats were euthanized, and their brains and other vital organs were removed, rinsed with saline solution, and photomicrographed in a variety of locations. Using the Image Pro-Plus software, the FITC signal that was present in brain tissue was analyzed. The findings are shown in Fig. 3.

### Glucose level measurement

An overnight fasting glucose level was determined by using blood samples taken from the tail at 8:00 to 8:30 in the morning on three separate occasions (one week after adaptation, after completing the intervention protocol, and 21 days following Aβ injection). In order to determine the levels of glucose in the blood, a glucose meter was utilized.

### Tissue preparation

Six entire hypothalamus from rats from each study group were extracted at the conclusion of the experiments, removed, and frozen in liquid nitrogen to be used later for qPCR gene expression and protein synthesis investigations.

The hypothalamus tissue of 6 rats from each group was extracted at the end of the experiments and frozen in liquid nitrogen to be used later for qPCR gene expression and protein synthesis research.

### RNA extraction and real-time PCR

Using NCBI Primer-BLAST, primers were created with rat specificity for hypothalamic cells. The software Gene Runner was used to create the IGF1, BDNF, and GLUT4 primers. The designed primers were blasted in order to verify accuracy and replicate only the genes' mRNA sequences. The sequences of the Real-Time PCR primers are shown in Table 1. Then, using RNX-PlusTM (Parstous, Iran), total RNA was extracted from the hypothalamus of all groups in accordance with the manufacturer's instructions. Then, a NanoDrop 2000c was used to confirm the quantity and purity of RNA (Eppendorf, Germany). Revert AidTM first-strand cDNA synthesis kit (Pishgam, Iran) was used to create cDNA from a 1000 ng sample of DNase-treated RNA using oligo (dT) primers. Master Mix and SYBR^®^ Green (Applied Biosystems, Life Technologies, and Paisley, UK) were used in Step One TM Applied Biosystems for PCR. Thermocycler parameters included 15 min at 95 °C, 40 cycles at 95 °C: 20 s, 60 °C: 30 s, and 72 °C: 30 s. The information was normalized using the housekeeping gene HPRT. The 2^−ΔΔCt^ technique was used to assess the relative expressions of the IGF1, BDNF, and GLUT4 genes in each of the groups under investigation^22^.

**Table 1:** Primer sequences used in Real-time PCR analysis.

Gene	Forward/reverse	Primer (5′ → 3′)
IGF1	F	CTGGTGGACGCTCTTCAGTT
	R	CCGGAAGCAACACTCATCCA
BDNF	F	GGCTCTCATACCCACTAAGATACATC
	R	CGGAAACAGAACGAACAGAAACAG
GLUT4	F	TCCAGTATGTTGCGGATGCTATG
	R	GTTTCAGGCACTCTTAGGAAGGT
HPRT	F	ATACAGGCCAGACTTTGTTGGA
	R	TCCACTTTCGCTGATGACACA

### ELISA assay

In a lysis solution (50 mM NaF, 20 mM Tris, 0.32 mM sucrose, 1 mM sodium-orthovanadate, 1 mM EDTA, 1 mM EGTA, and 1 mM phenyl methyl sulfonyl fluoride; Sigma-Aldrich, St. Louis, USA), the hypothalamus was homogenized (n = 3 animals per group (. In order to determine the levels of IGF1, BDNF, and GLUT4 protein in the tissue samples, a centrifugation process was conducted at a speed of 4000 rpm for a duration of 10 min to separate the serum. Subsequently, the samples were cleansed with 1X PBS to eliminate any surplus blood, homogenized in 20 mL of 1X PBS, and subsequently refrigerated at a temperature below – 20 °C for the duration of the night. Following two rounds of cryopreservation and subsequent thawing to disrupt the cell membranes, we subjected the homogenates to centrifugation at a speed of 5000 *g* for a duration of five minutes. We obtained the liquid portion, adhered to the guidelines provided by the manufacturer, and measured the levels of IGF1 protein using an ELISA Kit (MBS3808631, MyBioSource, USA), BDNF (MBS2019439, MyBioSource, USA), and GLUT4 (MBS765109, MyBioSource, USA). The optical density (O.D.) of each sample was quantified using a microplate reader (Stat Fax 4200, Awareness Technologies Inc.) configured to measure at a wavelength of 450 nm. The amounts of BDNF, GLUT4, and IGF1 in the samples were determined by comparing the optical density (O.D.) of the samples to the standard curve.

### Thioflavin-S staining

After deparaffinization and hydration, brain sections were incubated for 10 to 15 min at room temperature in filtered 1% aqueous Thioflavine-S (Sigma-T1892) before being washed three times with distilled water, glycerol, and PBS (Sigma-P4417) solution. The coverslip was then placed on the sample for fluorescent photography (under an Olympus microscope).

### Statistical analysis

Data from each trial were statistically analyzed using IBM SPSS Statistics 22 software, two-way ANOVA, and post-hoc analysis using the using the Tukey test. The cutoff for statistical significance was P ≤ 0.05. Measures of learning during the acquisition phase (such as travel distance and escape latency) were averaged for each animal over the course of each day in the Morris water maze test. Data were analyzed using a two- way repeated measures ANOVA with day as the within-subjects component and treatment (different groups) as the between-subjects factor to ascertain the differences between each day.

## Results

### Moderate treadmill exercise and insulin treatment reduced amyloid plaque load in hippocampus

**Figure 2:**
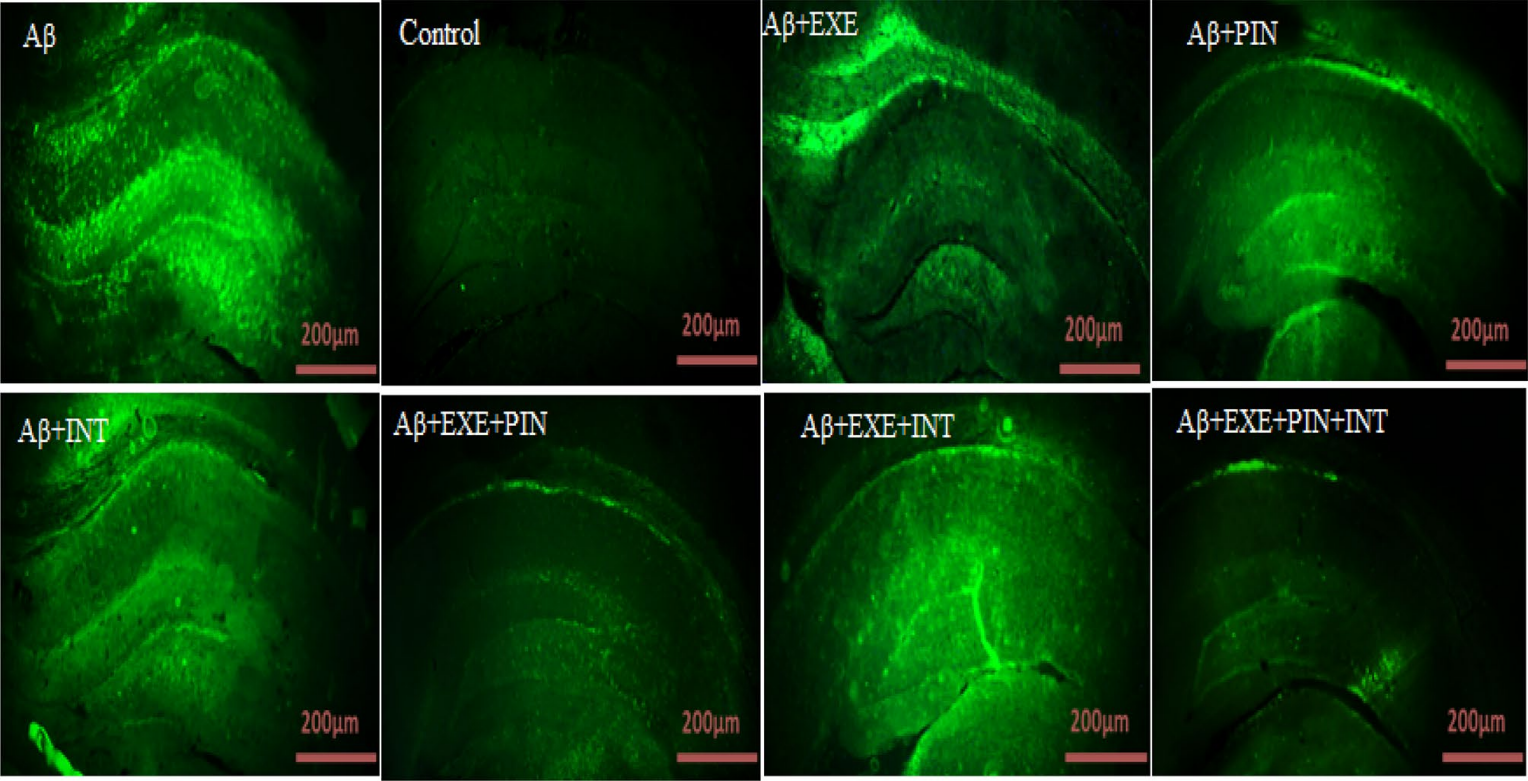
Hippocampal Thioflavin S staining. Using thioflavin S, the hippocampus tissue from the control, Aβ group, and treatment groups was stained. Magnification 400 ×; scale bars 200 µm.

**Figure 3:**
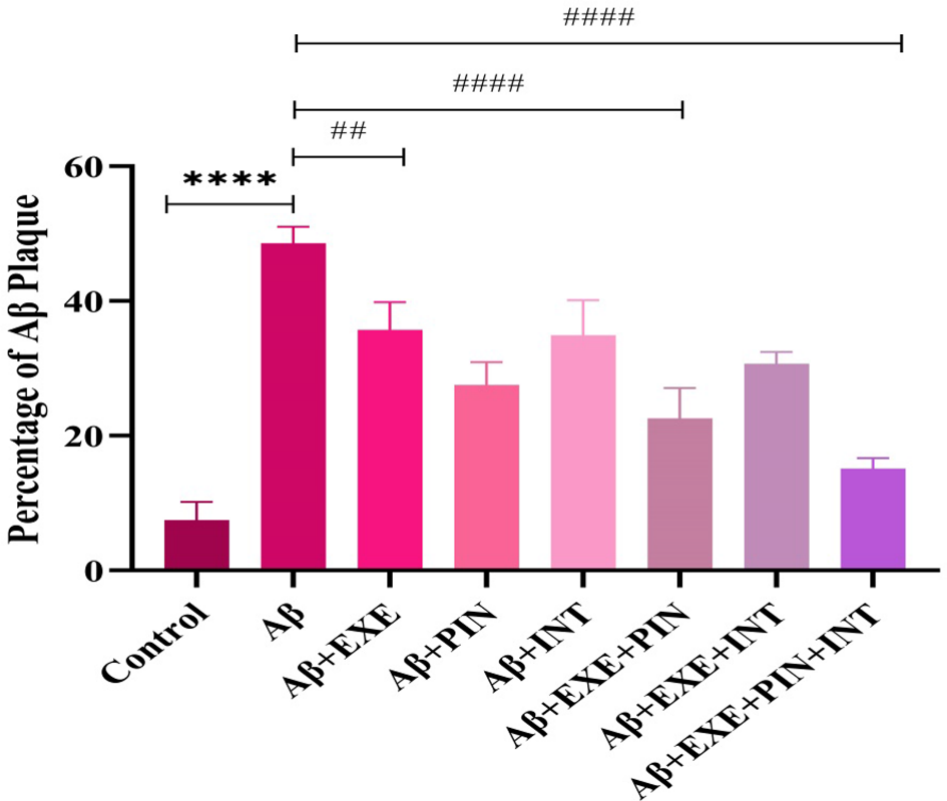
Hippocampal Thioflavin S staining. The control group's results revealed no amyloid deposits. In the Aβ, Aβ + EXE, Aβ + PIN, Aβ + INT, Aβ + EXE + PIN, Aβ + EXE + INT, and Aβ + EXE + PIN + INT groups, the Aβ deposits were visible in the images. The results were presented as the mean ± SEM (n = 3 in each group).

Utilizing the use of thioflavin S staining, we were able to determine the location of Aβ-fibrils in the hippocampus following ICV-Aβ injection. In comparison to the control group, 21 days following ICV-Aβ treatment, an aggregation of Aβ was shown to have occurred in the hippocampus. This finding is depicted in Fig. 2. In the hippocampus, a one-way ANOVA revealed a significant difference among the groups in the Aβ load (F_(7, 16)_ = 41.81, P = 0.0001). The staining results showed that the amount of beta amyloid accumulation was significantly higher in the group that had Aβ injection and did not receive any intervention (F_(7, 16)_ = 41.81, P < 0.0001). While exercising on a treadmill and receiving insulin significantly reduced the amount of Aβ plaque accumulation in the hippocampus compared to the Aβ group [Aβ + EXE (F_(7, 16)_ = 41.81, P = 0.005), Aβ + PIN (F_(7, 16)_ = 41.81, P = 0.0001), Aβ + INT(F_(7, 16)_ = 41.81, P = 0.003), Aβ + EXE + PIN (F_(7, 16)_ = 41.81, P = 0.0001), Aβ + EXE + INT (F_(7, 16)_ = 41.81, P = 0.0002), and Aβ + EXE + PIN + INT (F_(7, 16)_ = 41.81, P = 0.0001, Fig. 3).

### In-vivo optical imaging study

**Figure 4:**
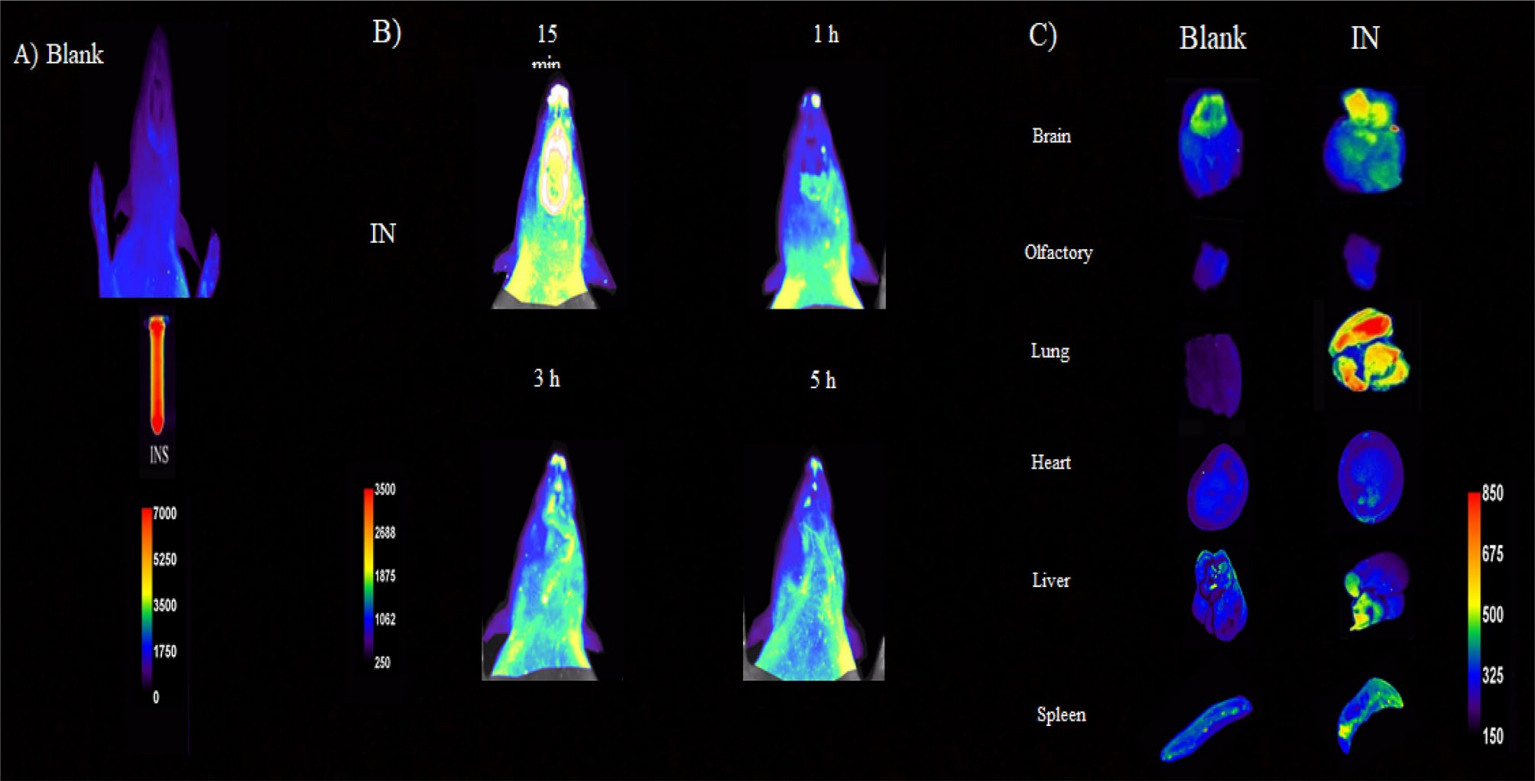
By using optical fluorescence imaging, one can observe the INS being taken in by Rat's nose. A) Shaved control rat, FITC-IN. B) Fluorescence images of the Brain, 15 min (top left row), 1 h (top right row), 3 h (bottom left row), and 5 h (bottom right row) after intranasal administration of FITC- IN suspension are shown. C) Optical imaging of the organs (5 h after injection, rats were slaughtered, and the organs were taken out). The images that were produced matched the outcomes of in vivo imaging.

The optical imaging system was used to test the fluorescent signal of FITC–CTI dispersed under the excitation at 510 nm and record it at 535 nm. This was done in order to investigate the delivery of IN to the brain and its distribution in the brain regions, as well as study the path of INS movement within the body. To demonstrate this point, Fig. 4A, the storage of FITC-IN showed a high fluorescence. Figure 4B, indicates that brain fluorescence is evident 15 min after FITC- IN injection in a way that is both clear and diffuse. The FITC-IN, on the other hand, showed a significant fluorescent signal in the neck and beneath the neck 1 h after injection, demonstrating that some of the FITC-IN had entered the pharynx. After three hours, there is a significant decrease in the amount of FITC emission in the brain. After 5 h, there was a greater reduction in the amount of FITC that was released in the brain. While fluorescence in the olfactory bubble nearly completely disappears, fluorescence in the lungs increases, suggesting that more FITC-IN may transfer from the nose to the lungs (Fig. 4C). In a separate study, a fluorescently labeled insulin nanoparticle, known as FITC-CTI, was used to determine the extent of insulin penetration into the brain. Several brain regions were examined at 15 min and 1 h after receiving FITC-CTI through the nose. The regions of the olfactory bulb and hippocampus contained the highest concentration of insulin nanoparticles^23^, which is in line with the results of the present study.

### Moderate training exercise and insulin treatment prevented the increase in blood glucose level caused by Aβ injection

**Figure 5:**
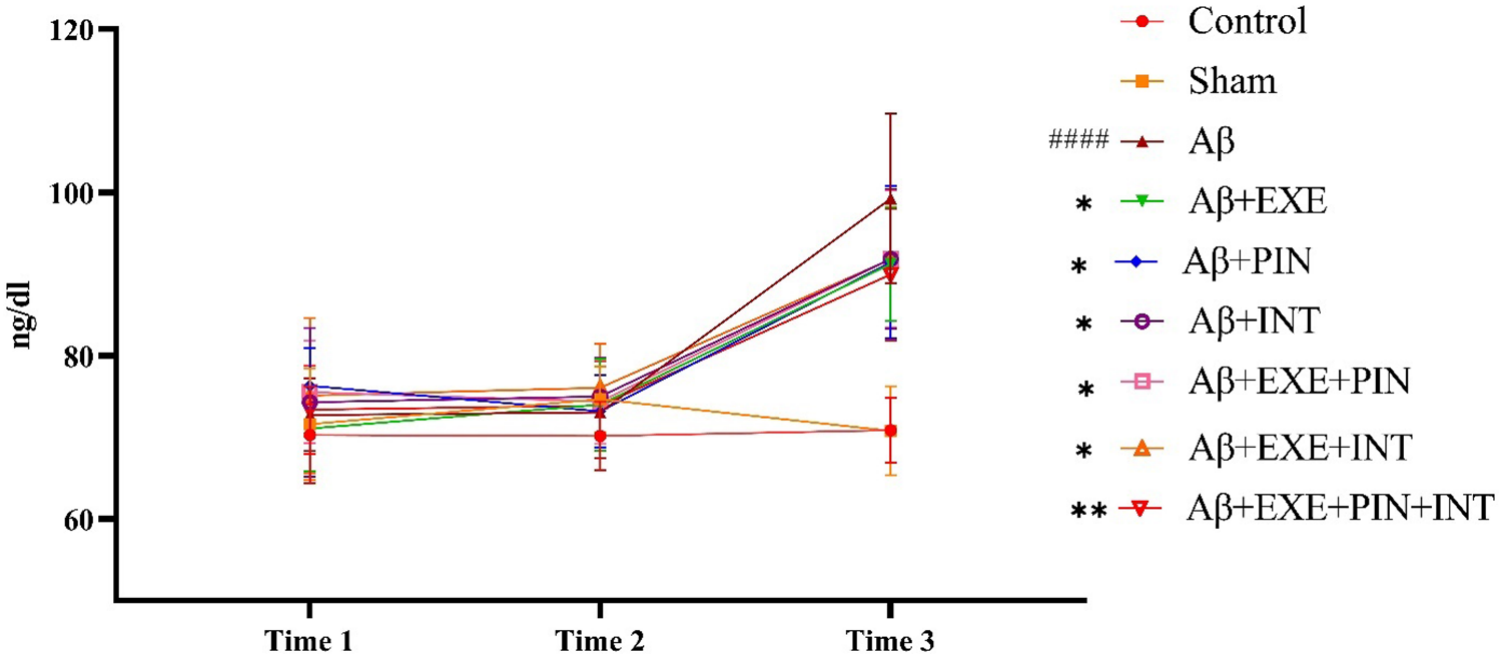
Blood glucose levels. According to the collected data, there were significant differences between the groups in the level of glucose in time3. The results were presented as the mean ± SEM (n = 16 in each group).

The statistical analysis revealed significant differences between the groups in the level of glucose at different times (F_(2, 405)_ = 216.6, p < 0.0001). In a two-way ANOVA with repeated measures, the results showed a significant difference between the groups in blood glucose levels (F_(8, 405)_ = 17.74, p < 0.0001). Also, time × treatment interaction was statistically significant (F_(16, 405)_ = 10.17, p < 0.0001). Post hoc Tukey multiple comparison tests were performed at times 3 and revealed that the group that received Aβ injections had a significantly greater level of glucose (p < 0.0001). Also, the groups that received moderate training exercise, insulin pretreatment, insulin treatment or a combination of both methods showed lower blood glucose levels than the Aβ group [Aβ + EXE (P = 0.02), Aβ + PIN (P = 0.02), Aβ + INT(P = 0.04), Aβ + EXE + PIN (P = 0.04), Aβ + EXE + INT (P = 0.04), and Aβ + EXE + PIN + INT (P = 0.002, Fig. 5).

### Moderate training exercise and insulin treatment improved learning and spatial memory

**Figure 6:**
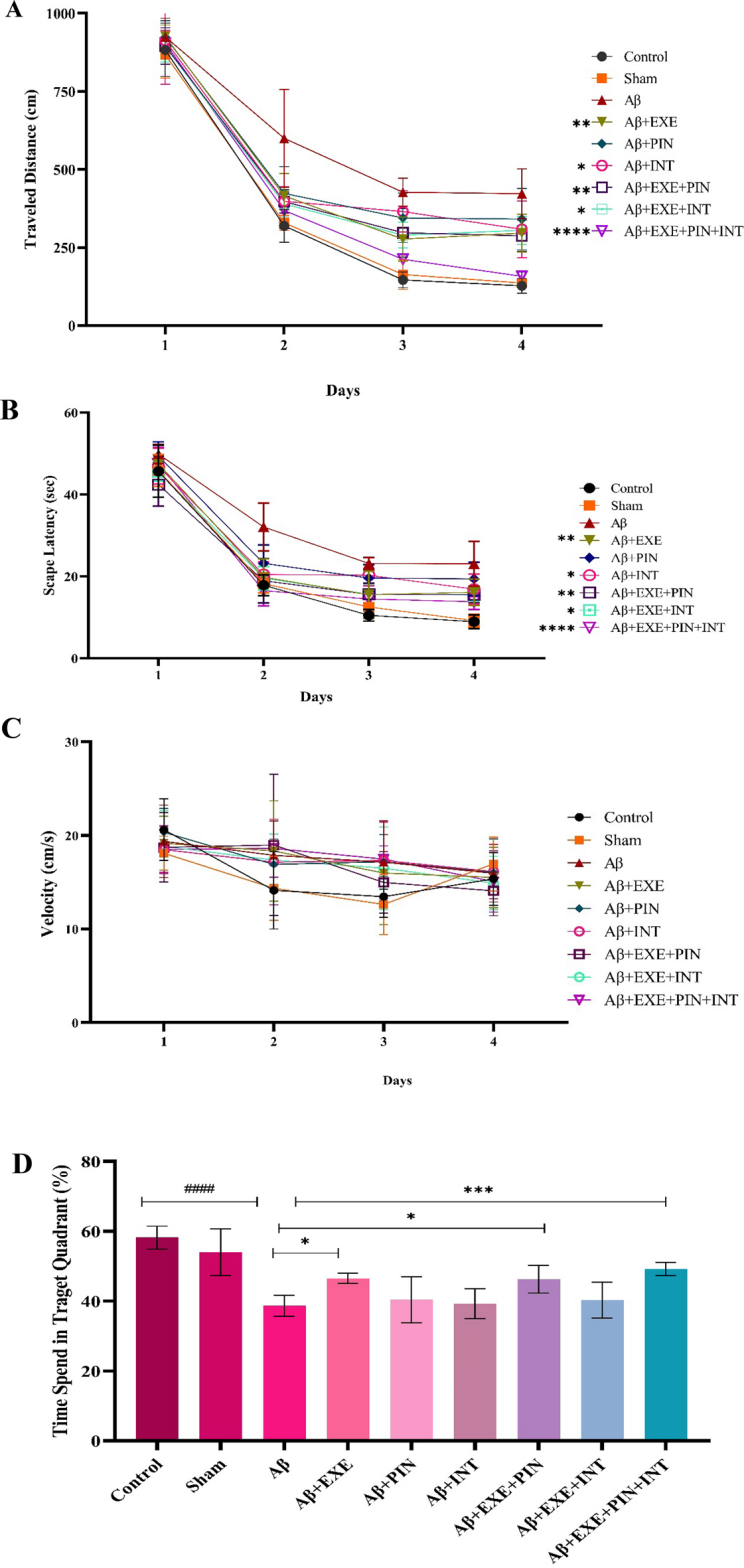
Test of MWM. Nine groups of adult Wistar rats were created at random. Spatial memory effects of ICV-Aβ/Saline dosing The MWM parameters for the several groups on the probing day were displayed in order from A to D. (**A**) Traveled distance: Rats receiving insulin and EXE showed a shorter travel distance to the hidden platform than rats receiving Aβ (P < 0.0001). (**B**) Escape Latency: The infusion of Aβ substantially reduced brain function. The EXE and IN therapy groups saw a considerable improvement in this condition (P < 0.001). (**C**) Velocity in the training phase, there was no significant difference in swimming speed during training between groups (p > 0.05). (**D**) Probe trial: In comparison to the group serving as the control, the A group's performance was subpar. In spite of this, the amount of time spent in the target zone significantly increased in the Aβ + EXE, Aβ + EXE + PIN, and Aβ + EX + PIN + INT groups (P < 0.0001). The Aβ impact appears to have been reversed by intranasal EXE and IN therapy. Results are shown as mean ± SEM (n = 8 in each group).

During the acquisition phase, learning was evaluated based on the decrease in escape latency and total distance traveled between days 1 to 4. Two-way ANOVA with repeated measures revealed that the main effect for day (within-subjects factor) was statistically significant [Fig. 6A,B; traveled distance: F_(3, 252)_ = 1450, P < 0.0001; escape latency: F_(3, 252)_ = 1265, P < 0.001], as was the main effect of treatment (between-subjects factor) [traveled distance: F_(8, 252)_ = 35.78, P < 0.001; escape latency: F_(8, 252)_ = 31.32, P < 0.001]. Also, day × treatment interaction was statistically significant [traveled distance: F_(24, 252)_ = 3.63, P = 0.0001; escape latency: F_(24, 252)_ = 2.87, P = 0.001]. The tukey post hoc multiple comparisons test for days 2, 3 and 4 showed a significantly longer traveled distance and escape latency for the Aβ injection group compared to the control group. The Tukey Post hoc multiple comparisons test was performed on days 1, 2, 3, and 4 and revealed that the group that received Aβ injections had significantly greater traveled distances and escape latencies when compared to the control group [Traveled distance: P < 0.0001 on day 2, P < 0.0004 on day 3, P < 0.006 on day 4; escape latency: P < 0.0001 on day 2, P < 0.0008 on day 3, P < 0.003 on day 4]. When compared to the Aβ group, the Aβ-EXE group considerably reduced both the traveled distance and the escape latency [Traveled distance: P > 0.001 on day 2, P < 0.0001 on day 3, P < 0.0001 on day 4; escape latency: P < 0.0001 on day 2, P > 0.0001 on day 3, P < 0.0001 on day 4]. Suggesting that running on a treadmill was able to prevent the impairment of spatial learning in rats that had been treated with Aβ. The findings also revealed a statistically significant difference in traveled distance and scape delay between the Aβ and Aβ + EXE + PIN + INT groups [Traveled distance: P > 0.001 on day 2, P < 0.0001 on day 3, P < 0.0001 on day 4; escape latency: P < 0.0001 on day 2, P > 0.0001 on day 3, P < 0.06 on day 4]. On day five of the probing test, the capacity for spatial memory was evaluated. The percentage of time spent in the targeted quadrant varied significantly between the groups, according to two-way ANOVA results (Fig. 6D; F_(8, 63)_ = 19.69, P < 0.0001). While this impairment was reduced in the Aβ + EXE, Aβ + EXE + PIN, and Aβ + EXE + PIN + INT groups, the results of a post hoc analysis using Tukey's HSD showed that Aβ_25-35_ injection significantly reduced the percentage of time spent in the target quadrant (P < 0.05). It was determined that there were no motor difficulties as a result of the administration of ICV-Aβ_25-35_ because there was no significant difference in swimming speed between the groups (Fig. 6C; F_(24, 252)_ = 1.12, p = 0.31).

### Moderate training exercise and insulin treatment increased the expression of IGF1, BDNF, and GLUT4 genes in the hypothalamus

**Figure 7:**
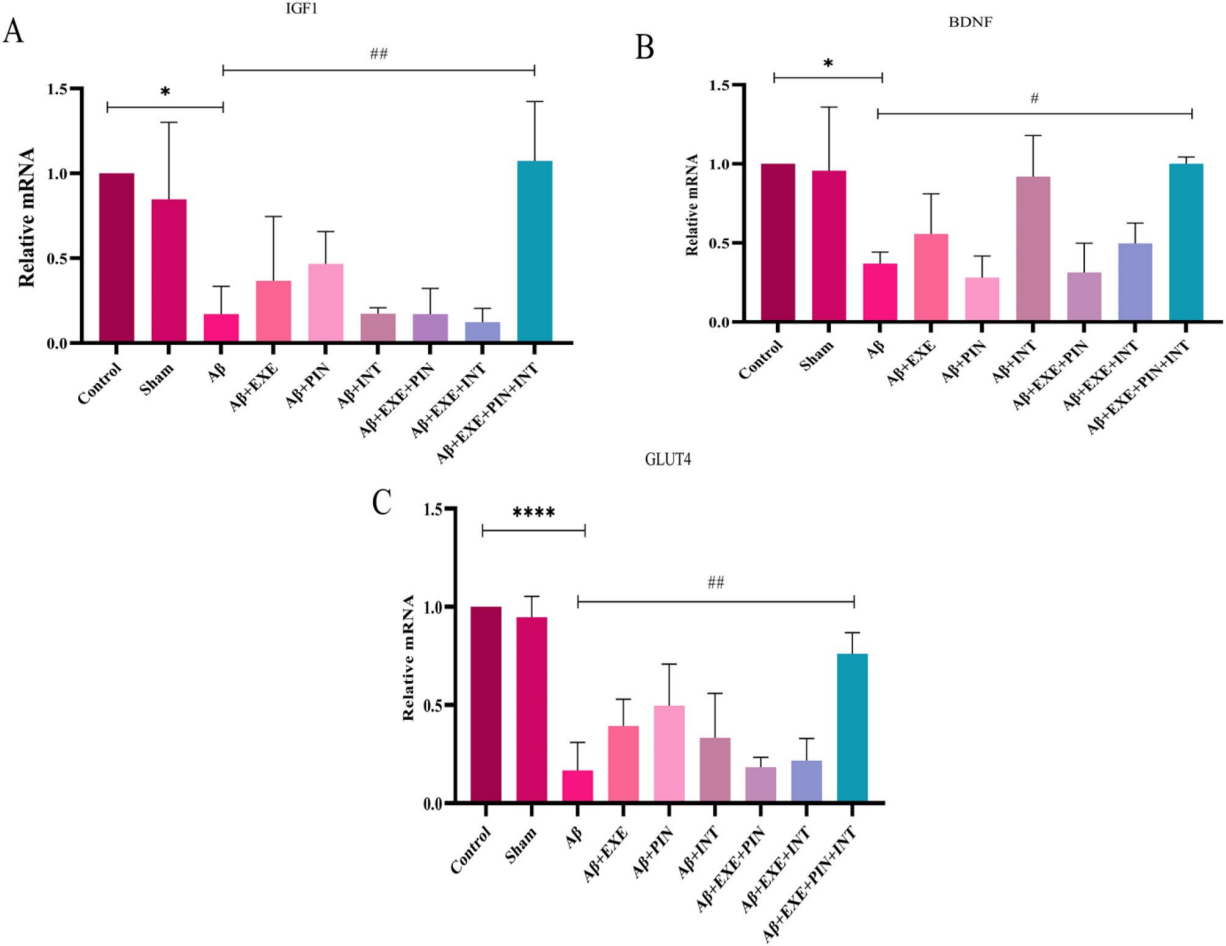
Gene expression of IGF1, BDNF, and GLUT4 in the hypothalamus. Aβ decreased the expression of IGF1, BDNF, and GLUT4 genes in the hypothalamus. Moderate training exercise along with receiving insulin as treatment and pretreatment significantly increased the mRNA levels of IGF1, BDNF, and GLUT4 in the hypothalamus of rats. Results are shown as mean ± SEM (n = 3 in each group).

The effects of interventions on the mRNA levels of IGF1, BDNF, and GLUT4 in the hypothalamus of all experimental groups are shown in Fig. 7. A two-way ANOVA revealed a significant difference among the groups in the mRNA levels of IGF1 (F_(8, 18)_ = 7.06, P = 0.0003), BDNF (F_(8, 18)_ = 7.03, P = 0.0003), and GLUT4 (F_(8, 18)_ = 16.58, P = 0.0001). Post-hoc comparisons showed that the Aβ_25–35_ injection significantly decreased the mRNA levels of IGF1 (F_(8, 18)_ = 7.06, P = 0.01), BDNF (F_(8, 18)_ = 7.03, P = 0.02), and GLUT4 (F_(8, 18)_ = 16.58, P = 0.0001) in the hypothalamus of rats. While moderate training exercise along with receiving insulin as treatment and pretreatment significantly increased the mRNA levels of IGF1 (F_(8, 18)_ = 7.06, P = 0.007), BDNF (F_(8, 18)_ = 7.03, P = 0.02), and GLUT4 (F_(8, 18)_ = 16.58, P = 0.001) in the hypothalamus of the Aβ group rat. Aβ + EXE + PIN + INT increased compared to the Aβ group. Moreover, the results demonstrated that there was no significant difference in the mRNA levels of IGF1 (F_(8, 18)_ = 7.06, P = 0.9), BDNF (F_(8, 18)_ = 7.03, P = 0.9), and GLUT4 (F_(8, 18)_ = 16.58, P = 0.9) between the control and Sham (saline) groups (Fig. 7A–C).

### Moderate training exercise and insulin treatment caused an increase in the levels of IGF1, BDNF, and GLUT4 proteins in the hypothalamus

**Figure 8:**
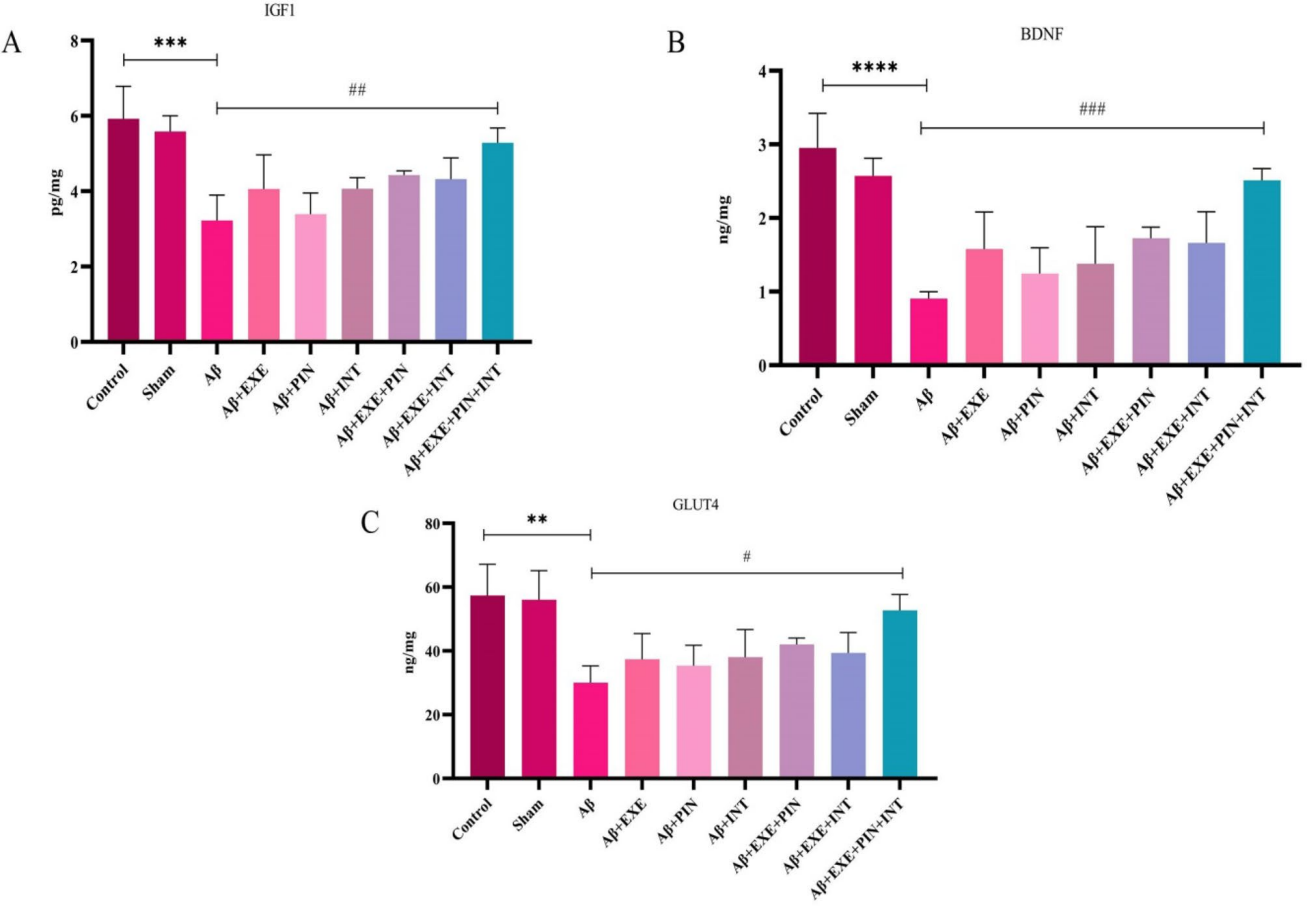
ELISA analysis of IGF1, BDNF, and GLUT4 in the hypothalamus. Aβ decreased levels of IGF1, BDNF, and GLUT4 in the hypothalamus of a rat model of AD. Rat treated with moderate training exercise and insulin treatment showed a higher level of IGF1, BDNF, and GLUT4 proteins in the hypothalamus. Measured by the ELISA kit. Moderate training exercise and insulin treatment significantly increased the level of IGF1 (F_(8, 18)_ = 7.72, P = 0.009), BDNF (F_(8, 18)_ = 11.20, P = 0.008), and GLUT4 (F_(8, 18)_ = 5.59, P = 0.02) proteins in the hypothalamus of group B rats. Results are shown as mean ± SEM (n = 3 in each group).

In the hypothalamus, a two-way ANOVA revealed a significant difference among the groups in the levels of IGF1 (F_(8, 18)_ = 7.72, P = 0.0002), BDNF (F_(8, 18)_ = 11.20, P = 0.0001), and GLUT4 (F_(8, 18)_ = 5.59, P = 0.001). Elisa results indicated that protein levels of IGF1 (F_(8, 18)_ = 7.72, P = 0.0006), BDNF (F_(8, 18)_ = 11.20, P = 0.0001), and GLUT4 (F_(8, 18)_ = 5.59, P = 0.004) in Aβ group were significantly lower than that of control group, while moderate treadmill exercise and received insulin treatment significantly increased the protein levels of IGF1 (F_(8, 18)_ = 7.72, P = 0.009), BDNF (F_(8, 18)_ = 11.20, P = 0.008), and GLUT4 (F_(8, 18)_ = 5.59, P = 0.02) in the hypothalamus of Aβ + EXE + PIN + INT rats compare to Aβ rats (Fig. 7A–C). Moreover, moderate training exercise alone and insulin treatment did not significantly increase the protein levels of IGF1, BDNF, and GLUT4 in the hypothalamus (P > 0.05) (Fig. 8A–C).

## Discussion

According to our findings, 3 weeks after Aβ_25–35_ injection, compared to vehicle- or control-injected rats, spatial learning and memory were reduced. This was demonstrated by an increase in trip distance and escape latency as well as a decrease in the time spent in the targeted quadrant in the MWM test. AD is characterized by a gradual decline in a person's cognitive abilities, particularly their recall and ability to learn^24^. In this study, we used an established model that involved the intra-cerebroventricular injection of Aβ_25–35_ fibrils. This impaired the rats' ability to acquire and remember spatial information in the MWM test 21 days after the injection, confirming and expanding upon previous reports^16,25^. Animal research has demonstrated that exercise boosts levels of neurotrophic factors, which in turn increase neurogenesis, synaptic plasticity, antioxidant capacity, and angiogenesis in models of Alzheimer's disease (AD). This has the potential to improve cognitive function and memory impairment in AD patients^26^. Also, thioflavin S staining showed that amyloid plaques were formed in the hippocampus 21 days after the injection^13^. Additionally, it is possible that the reduction in plaque burden and soluble Aβ levels in the hippocampus that occurs as a result of moderate training exercise and intranasal insulin is responsible for the improvement in impaired cognition that occurs^15^. In this context, there is a growing body of evidence that suggests that the soluble and oligomer forms of Aβ play a significant role in the initiation of cognitive impairments. This is achieved by specifically targeting synapses and altering synaptic signaling pathways^27^. Deficits in long term potentiation (LTP) and synaptic loss in AD may be caused by an increase in soluble Aβ before amyloid plaques form^28^. A theory that could be used to explain the related mechanism for the exercise-induced reduction of Aβ in the hippocampus of Alzheimer's disease (AD) observed in the current study could be that its clearance is altered^29^. The insensitivity of hippocampal IRs and the reduced phosphorylation of insulin downstream signaling molecules are the hallmarks of hippocampus insulin resistance. Research has demonstrated a robust correlation between hippocampus insulin resistance and AD pathogenesis, encompassing tau hyperphosphorylation and Aβ aggregation. Insulin signaling causes tau hyperphosphorylation and Aβ deposition directly, while brain insulin resistance lowers IDE (insulin degrading enzyme) levels. It is not unexpected that the disruption of insulin signaling in the hippocampus has a significant impact on LTP establishment and cognitive function, since some of the effects of insulin in the hippocampus are linked to the establishment of synaptic plasticity, neuronal growth, antioxidant protection, and, to some extent, control of glucose metabolism.

When insulin (INS) binds to its receptor, the IRS-1/PI3-K/Akt cascade is triggered. Activation of mTOR/S6K signaling results in increased production of proteins in the synapse, including PSD-95 and NMDAR, AMPAR, and GABAAR receptors. Akt facilitates the entry of Na + and Ca2 + into the synapse as well as the uptake of glucose by the insulin-dependent glucose transporter GLUT4 and the glutamate receptors NMDAR, AMPAR, and GABAAR into the membrane. Insulin signaling also prevents tau phosphorylation by suppressing GSK3β activity. All things considered, improved insulin-promoted protein synthesis and transport form, glutamate transmission, synaptic metabolism, and neuron survival and proliferation result in the formation of LTP, memory, and learning^30–32^. Moreover, oxidative stress and neuroinflammation brought on by insulin resistance have a role in controlling the pathogenic advancement of AD^33^.

On the other hand, it has been demonstrated that engaging in regular exercise and intranasal insulin is connected with increased cognitive capacity in AD condition^23,34^. We demonstrated that treadmill exercise and intranasal insulin enhance cognitive performance in normal rats, which is consistent with prior research (increase in the percentage of time spent in the targeted quadrant and number of times crossing the platform)^15,16,23^. In the past, researchers have demonstrated that giving mice insulin through their noses can enhance their defective spatial memory as well as their cognitive impairment and learning^23^. Surprisingly, rats that received insulin and exercise combined in their program and rats that only exercised had better learning and spatial memory performance (decreased escape latency and distance traveled, increased percentage of time spent in the target quadrant, and the number of platform crossings) compared to Aβ_25-35_-treated rats, indicating the role of treadmill exercise and intranasal insulin in improving cognitive performance even after developing similar pathology. Previous results showed that both exercise and intranasal insulin treatment increased PGC-1α (Peroxisome proliferator-activated receptor-gamma coactivator- 1alpha) protein levels as well as mitochondrial proteins and mitochondrial ATP production, providing evidence to support a direct role of exercise and insulin in increasing brain mitochondrial biogenesis and ATP production^16,35^.

Exercise training and insulin are both known to improve cognitive function, but the exact mechanism by which these occur is not completely understood. It is believed that the pathology of Alzheimer's disease usually occurs in the cortex and hippocampus, the areas responsible for coordination and spatial memory, and slowly spreads throughout the central nervous system. Evidence from a study shows that metabolic deficits and disorders occur before Alzheimer's pathology^36^. Research suggests Alzheimer's disease may be linked to a 10–12% reduction in the volume of the hypothalamus^37–40^. The fact that metabolically significant changes take place before the buildup of amyloid plaques and tangles-enriched neurodegeneration suggests that Alzheimer's disease may also be a metabolic disorder. AD is not just a neurodegenerative illness. The lack of a compensatory increase in adipose tissue may have been caused by an increase in metabolic rates that more than offset the effect of the higher calorie consumption. This indicates that metabolic abnormalities are already present in the 3xtg AD mouse model at an early stage, long before any of the classic symptoms of AD, such as the formation of amyloid plaques in the brain^41^. It's possible that these metabolic irregularities play a part in the development of AD. Alterations in energy metabolism, oxidative damage, and a decreased capacity of the organism and its cells to handle stress are all factors in the aging process and the diseases it is linked with. We suggest that particular signaling pathways in the brain may be significant determinants of health as one age, and we base this hypothesis on previous research. IGF1, BDNF, and GLUT4 are examples of specific signaling modalities that can stimulate other signaling modalities in neurons^42–45^. IGF1 maintains the proliferation of numerous peripheral and central cells and is essential for metabolism and growth^7^. An essential part of the hypothalamic system that regulates energy balance is BDNF^10^. Also, the activity of GLUT4 in the brain is necessary for the preservation of glucose homeostasis, the generation of energy, and the survival of neuronal cells^46^. According to our findings, rats given amyloid had lower levels of IGF1, BDNF, and GLUT4 gene expression in the hypothalamus than animals in the control group. In this regard, compared to the amyloid group, rats that underwent treadmill exercise for 4 weeks in addition to intranasal insulin pretreatment and therapy showed higher levels of IGF1, BDNF, and GLUT4 gene expression in the hypothalamus. Also, our findings demonstrated that the decrease in the protein levels of IGF1, BDNF, and GLUT4 in the Aβ-treated rats is prevented by treadmill exercise, pretreatment, and treatment with insulin.

Insulin-like growth factor-1 (IGF-1) has been hypothesized to be associated with both physical activity and cognitive function^47,48^. In the process of neurogenesis, this component appears to play a crucial function^49,50^. IGF-1 has a further connection to the development and differentiation of neurons in the brain since it plays a function in the clearance of β-amyloid (Aβ) and tau hyperphosphorylation^51^. Additionally, it appears that insulin resistance is accompanied by IGF-1 resistance and is connected with a malfunction in the activity of IRS-1^52^. The PI3K/Akt pathway modulates glucose transporters (GLUTs) (GLUT1, GLUT2, GLUT3, and GLUT4) to regulate brain glucose absorption. GLUTs allow glucose to enter astrocytes and neurons across the blood–brain barrier. Insulin resistance, decreased GLUT-dependent brain glucose uptake, reduced glycolytic flux, and poor mitochondrial function have been linked to AD progression in observational studies^53,54^. In addition, research has demonstrated that physical activity reduces inflammation in the brains of people with Alzheimer's disease, which in turn improves insulin resistance-induced glucose metabolic dysfunction.

The fact that BDNF may be regulated by IGF-1 is one hypothesis that could be considered one of the possibilities. In the context of exercise-related reduction of Alzheimer's disease pathogenesis, the activation of BDNF plays a significant role^16^. Through modulation by α-, β-, and γ-secretases as well as GSK3, BDNF can directly alter intracellular Aβ synthesis, tau phosphorylation, and neurogenesis^55,56^.

Inflammation is one of the potential effective causes of glucose metabolism disorders and cognitive disorders. In a conceptual model put forth by Cai and coworkers (2015), hypothalamic microinflammation is viewed as a common cause of metabolic syndrome and aging^57^. The hypothalamus is responsible for coordinating the activities of neural circuits and neuroendocrine hormones, which together maintain proper nutrient and energy homeostasis. Overeating and inactivity cause alterations in the hypothalamus that resemble inflammation^10^. In fact, excessive nutrition combined with a lack of exercise might influence the course that inflammation takes, leading to an increase in variables such as IL-6 and TNF-α as well as reactive oxygen species (ROS). When these pathways are activated, insulin resistance develops, which in turn causes an increase in the level of glucose in the blood^58^. Exercise and intranasal insulin may alter how mediators of the oxidative-inflammatory cascade function by raising levels of IGF1, BDNF, and GLUT4 in the hypothalamus. In fact, the modification of this pathway, which may be the result of increasing the number of proteins mentioned above, leads to an improvement in glucose metabolism and blood glucose levels. According to our findings, compared to the Aβ group, blood glucose levels are improved by exercise and intranasal insulin.

## Conclusion

The current research demonstrates that intraventricular injection of Aβ_25-35_ results in impairment in learning and spatial memory, as well as a reduction in the expression of IGF1, BDNF, and GLUT4 in the hypothalamus of adult male rats. Moderate training exercise and insulin treatment improved spatial learning and memory, which was associated with decreased Aβ plaque load in the hippocampus, increased gene expression, and increased levels of IGF1, BDNF, and GLUT4 proteins in the hypothalamus in Aβ_25-35_ treated rats. The results of the present study show that the improvement of memory and spatial learning has probably affected the metabolic factors of the hypothalamus through changing the expression of genes and proteins of IGF1, BDNF, and GLUT4. More studies are needed to find out the exact mechanism.

## Data Availability

The datasets generated and/or analysed during the current study are available in the [Mendeley Data] repository, [R12700″, Mendeley Data, V1, 10.17632/dkyn2bcvb9.1].
